# Time-Engineered Hydrothermal Nb_2_O_5_ Nanostructures for High-Performance Asymmetric Supercapacitors

**DOI:** 10.3390/nano16030173

**Published:** 2026-01-27

**Authors:** Rutuja U. Amate, Mrunal K. Bhosale, Aviraj M. Teli, Sonali A. Beknalkar, Hajin Seo, Yeonsu Lee, Chan-Wook Jeon

**Affiliations:** 1 School of Chemical Engineering, Yeungnam University, 280 Daehak-ro, Gyeongsan 38541, Republic of Korea; 2 Division of Electronics and Electrical Engineering, Dongguk University-Seoul, Seoul 04620, Republic of Korea

**Keywords:** Nb_2_O_5_ nanostructures, hydrothermal time engineering, pseudocapacitance, diffusion-controlled charge storage, asymmetric supercapacitor, energy storage materials

## Abstract

Precise control over nanostructure evolution is critical for optimizing the electrochemical performance of pseudocapacitive materials. In this work, Nb_2_O_5_ nanostructures were synthesized via a time-engineered hydrothermal route by systematically varying the reaction duration (6, 12, and 18 h) to elucidate its influence on structural development, charge storage kinetics, and supercapacitor performance. Structural and surface analyses confirm the formation of phase-pure monoclinic Nb_2_O_5_ with a stable Nb^5+^ oxidation state. Morphological investigations reveal that a 12 h reaction time produces hierarchically organized Nb_2_O_5_ architectures composed of nanograin-assembled spherical aggregates with interconnected porosity, providing optimized ion diffusion pathways and enhanced electroactive surface exposure. Electrochemical evaluation demonstrates that the NbO-12 electrode delivers superior pseudocapacitive behavior dominated by diffusion-controlled Nb^5+^/Nb^4+^ redox reactions, exhibiting high areal capacitance (5.504 F cm^−2^ at 8 mA cm^−2^), fast ion diffusion kinetics, low internal resistance, and excellent cycling stability with 85.73% capacitance retention over 12,000 cycles. Furthermore, an asymmetric pouch-type supercapacitor assembled using NbO-12 as the positive electrode and activated carbon as the negative electrode operates stably over a wide voltage window of 1.5 V, delivering an energy density of 0.101 mWh cm^−2^ with outstanding durability. This study establishes hydrothermal reaction-time engineering as an effective strategy for tailoring Nb_2_O_5_ nanostructures and provides valuable insights for the rational design of high-performance pseudocapacitive electrodes for advanced energy storage systems.

## 1. Introduction

The ongoing transition toward electrified transportation, decentralized renewable energy generation, and compact multifunctional electronics has fundamentally altered the operational requirements of modern energy storage systems [1]. In practical environments, these systems are routinely exposed to rapid power fluctuations, short-duration charging events, and continuous cycling under high current loads [2]. Conventional battery technologies, while effective for high energy storage, often struggle to maintain stability and efficiency under such dynamic conditions, leading to accelerated degradation, thermal stress, and reduced power responsiveness [3]. These limitations underscore the urgent need for alternative energy storage solutions that can deliver rapid charge–discharge capability while retaining structural integrity and long-term reliability [4]. Supercapacitors have emerged as key components in this landscape due to their ability to deliver high power density, rapid charge–discharge response, and exceptional cycling stability [5]. However, their limited energy density remains a persistent obstacle to broader adoption in advanced energy systems. Improving the energy storage capability of supercapacitors necessitates electrode materials that can support fast surface or near-surface redox reactions while maintaining efficient pathways for both electron transport and ion diffusion [6]. As a result, the development of pseudocapacitive materials with optimized structural and electrochemical characteristics has become a central challenge in supercapacitor research [7]. Among the various classes of pseudocapacitive materials, transition metal oxides such as MnO_2_, NiO, V_2_O_5_, Co_3_O_4_, and RuO_2_ have been extensively investigated due to their multiple oxidation states and high theoretical charge storage capacity [8]. Nevertheless, many of these materials suffer from slow ion transport, poor electrical conductivity, and structural instability during prolonged cycling, which limit their performance under high-rate operation. In this context, niobium oxide (Nb_2_O_5_) has attracted growing attention owing to its distinctive charge storage behavior and structural resilience. Charge storage in Nb_2_O_5_ is governed by reversible Nb^5+^/Nb^4+^ redox reactions accompanied by rapid ion insertion into near-surface lattice sites, a process that proceeds without significant crystallographic phase transformation [9]. This intercalation-dominated pseudocapacitive mechanism enables fast kinetics and minimal volume change, making Nb_2_O_5_ particularly suitable for high-power energy storage applications [10].

Recent studies have highlighted the advantages of Nb_2_O_5_-based electrodes for supercapacitor applications. Wan et al. identified Nb_2_O_5_ as a promising energy storage material due to its high specific capacity and resistance to phase transition; however, they also noted that the facile formation of niobic acid complicates controlled growth and limits electrical conductivity and structural stability, particularly for low-dimensional architectures [11]. Vicentini et al. demonstrated that Nb_2_O_5_ nanoparticle-decorated graphene electrodes exhibited enhanced pseudocapacitive contribution, suppressed electrolyte decomposition, and an extended operating voltage window in aqueous supercapacitors, resulting in increased energy and power densities along with reduced equivalent series resistance [12]. Similarly, Nagaraju et al. reported Nb_2_O_5_/graphene nanocomposites synthesized without surfactants, achieving a high specific capacitance of 633 F g^−1^ and excellent cycling stability with 99.3% retention after 5000 cycles, attributed to improved charge transport and effective utilization of active sites [13]. While these studies underscore the potential of Nb_2_O_5_, challenges associated with controlled growth and performance optimization through synthesis parameters remain insufficiently addressed. Hydrothermal engineering has emerged as a powerful strategy for controlling the physicochemical properties of pseudocapacitive electrodes because it enables precise regulation of nucleation density, crystal growth rate, defect formation, and morphology evolution under mild aqueous conditions. In particular, reaction-time modulation plays a decisive role in determining the final electrode architecture by governing dissolution–recrystallization behavior and Ostwald ripening, thereby influencing crystallinity, particle coarsening, pore connectivity, and active surface exposure. Short hydrothermal durations often yield underdeveloped structures with limited electroactive accessibility, whereas excessive reaction time can cause particle agglomeration and densification, reducing electrolyte penetration and increasing ion-diffusion resistance. Therefore, optimizing hydrothermal duration provides a practical pathway to simultaneously balance structural uniformity, electrolyte-accessible porosity, and fast charge-transfer/ion-transport kinetics, which are essential for achieving high-rate pseudocapacitive performance. Despite this significance, most Nb_2_O_5_-based supercapacitor studies mainly focus on compositional modifications, while systematic investigations correlating reaction-time-driven structural evolution of Nb_2_O_5_ with electrochemical kinetics and device-level performance remain comparatively limited, highlighting a clear research gap addressed in this work. Hydrothermal synthesis provides a versatile platform for regulating the growth and structural evolution of metal oxides under mild conditions. Reaction time, in particular, plays a decisive role in governing nucleation, growth kinetics, and surface development. Inadequate reaction durations can yield poorly developed structures, whereas excessive growth may result in agglomeration and diminished electrochemical accessibility [14]. Despite its importance, the influence of hydrothermal reaction time on Nb_2_O_5_ electrode performance has not been clearly established [15].

In this work, Nb_2_O_5_ nanostructures are synthesized via a hydrothermal route with controlled reaction times of 6, 12, and 18 h, yielding samples denoted as NbO-6, NbO-12, and NbO-18, respectively. The influence of reaction time on structural evolution, charge transport behavior, and electrochemical performance is systematically examined. Furthermore, the Nb_2_O_5_ electrode is employed as the positive electrode in an asymmetric supercapacitor to evaluate its practical energy storage performance. This study provides clear insight into time-dependent structural control of Nb_2_O_5_ and establishes a rational framework for designing high-performance pseudocapacitive electrodes for advanced supercapacitor applications.

## 2. Experimental Section

### 2.1. Materials

Niobium pentachloride (NbCl_5_, Sigma-Aldrich, 99%, St. Louis, MO, USA) and oxalic acid (C_2_H_2_O_4_, Alfa Aesar, 98%, Ward Hill, MA, USA) were used as received. Polyvinylidene fluoride (PVDF, Sigma-Aldrich, St. Louis, MO, USA), N-methyl-2-pyrrolidone (NMP, Sigma-Aldrich, St. Louis, MO, USA), and conductive carbon black were used for electrode preparation. Deionized (DI) water was used throughout. Nickel foam (1 × 2 cm^2^) served as the current collector.

### 2.2. Hydrothermal Synthesis of Nb_2_O_5_ Nanostructures

**Figure 1:**
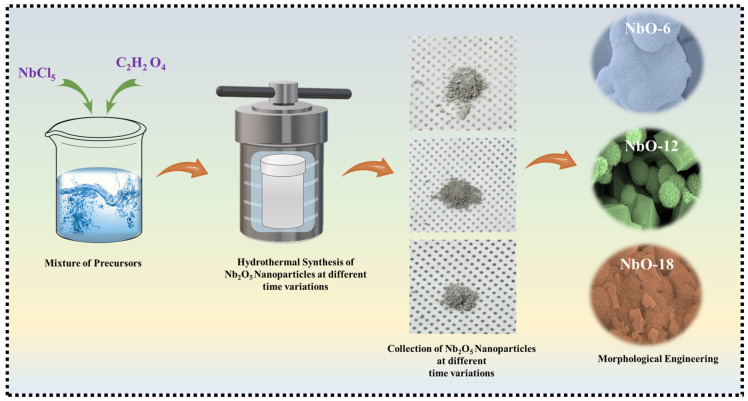
Schematic illustration of the time variation hydrothermal process used for synthesizing Nb_2_O_5_ electrodes.

Nb_2_O_5_ powders were synthesized via a hydrothermal method. First, 0.02 M NbCl_5_ was dissolved in 40 mL of DI water and stirred for 10 min. Separately, 0.2 M C_2_H_2_O_4_ was dissolved in 20 mL of DI water and stirred for 10 min. The two solutions were then combined and stirred together for 20 min to obtain a homogeneous precursor solution. The resulting solution was transferred into a 100 mL Teflon-lined stainless-steel autoclave and heated at 180 °C for 6, 12, or 18 h. After naturally cooling to room temperature, the powders were collected by centrifugation, washed several times with DI water and ethanol to remove impurities, and dried at 60 °C overnight. The dried powders were then annealed at 600 °C for 2 h in air to obtain crystalline Nb_2_O_5_ with improved structural stability. Figure 1 schematically depicts the stepwise hydrothermal synthesis route adopted in this work, illustrating the controlled formation and evolution of Nb_2_O_5_ nanostructures under varied reaction durations.

### 2.3. Electrode Fabrication

For electrochemical testing, the annealed Nb_2_O_5_ powders were mixed with conductive carbon black and PVDF binder in an 80:10:10 weight ratio. NMP was added to form a uniform slurry (2 mg/cm^2^), which was then coated onto pre-cleaned nickel foam (1 × 1 cm^2^), using a manual doctor-blade method, without any pressure applied. The coated electrodes were dried at 80 °C under vacuum for 12 h and lightly pressed to improve adhesion. The electrodes were directly used in a three-electrode configuration for electrochemical measurements.

## 3. Material Characterization and Electrochemical Measurements

The structural, morphological, and surface chemical properties of the synthesized Nb_2_O_5_ samples were systematically investigated using complementary characterization techniques. X-ray diffraction (XRD) analysis was carried out using a PANalytical diffractometer (Birmimgham, UK) equipped with Cu Kα radiation (λ = 1.5406 Å) to identify the crystalline phase and structural evolution as a function of hydrothermal reaction time. Diffraction patterns were recorded over an appropriate 2θ range to ensure reliable phase identification. The surface morphology and microstructural features of the Nb_2_O_5_ powders were examined by field-emission scanning electron microscopy (FE-SEM, HITACHI S-4800 HITACHI, Tokyo, Japan). Prior to imaging, the samples were sputter-coated with a thin platinum layer to suppress surface charging and improve image clarity. Elemental composition and spatial distribution of niobium and oxygen were analyzed using energy-dispersive X-ray spectroscopy (EDS) coupled with the FE-SEM system. X-ray photoelectron spectroscopy (XPS, K-Alpha, Thermo Scientific, Eastleigh, UK) measurements were performed to analyze the surface chemical states and oxidation environment of niobium and oxygen in the sample (XPS measurements were performed on the annealed Nb_2_O_5_ powder). Electrochemical measurements were conducted using a Biologic potentiosta (WBCS3000 (Gières, France)) in a three-electrode configuration. The Nb_2_O_5_-coated nickel foam served as the working electrode, while platinum foil and an Ag/AgCl electrode were used as the counter and reference electrodes, respectively. All electrochemical tests were carried out in 2 M KOH aqueous electrolyte at room temperature. Cyclic voltammetry (CV) was employed to evaluate charge storage behavior and redox activity, whereas galvanostatic charge–discharge (GCD) measurements were performed to assess capacitive performance at different current densities. Electrochemical impedance spectroscopy (EIS) was used to investigate charge-transfer resistance and ion transport characteristics. Cycling stability tests were conducted to examine the durability of the electrode materials under prolonged operation.

## 4. Results and Discussion

### 4.1. X-Ray Diffraction Elucidation

**Figure 2:**
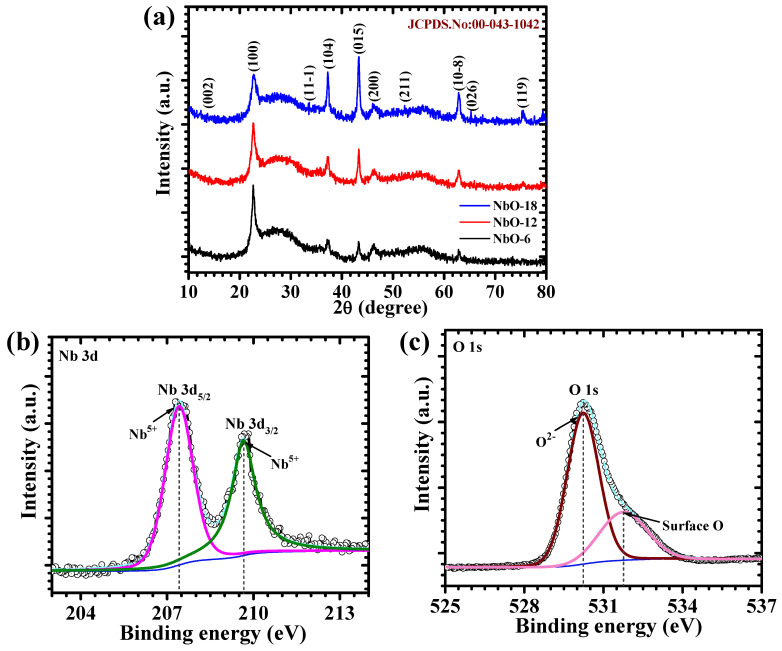
(**a**) XRD pattern of Nb_2_O_5_ electrodes (JCPDS reference card (No. 00-043-1042)); High-resolution XPS spectra of the NbO-12 sample: (**b**) Nb 3d, and (**c**) O 1s.

Figure 2a shows the XRD patterns of NbO-6, NbO-12, and NbO-18 samples, measured at appropriate diffraction range of 10–80°. All diffraction peaks can be well indexed to the monoclinic phase of Nb_2_O_5_, in good agreement with the standard JCPDS card No. 00-043-1042, confirming the successful formation of phase-pure niobium oxide without any detectable secondary phases or impurities. The diffraction reflections appearing at 2θ values of approximately 22.6°, 28.4°, 36.6°, 39.8°, 46.2°, 49.3°, 55.2°, 62.7°, 65.4°, and 72.8° correspond to the (100), (002), (11-1), (104), (015), (200), (211), (10-8), (026), and (119) crystallographic planes of monoclinic Nb_2_O_5_, respectively. The comparison among the three samples reveals that increasing the hydrothermal reaction time leads to a gradual enhancement in peak definition and intensity, indicating improved structural ordering. NbO-6 exhibits relatively broader and weaker diffraction peaks, suggesting incomplete crystallization at shorter reaction duration. The relatively broader diffraction peaks observed for the NbO-6 sample can be attributed to the combined effects of limited crystallite size, residual lattice microstrain associated with early-stage nucleation, and a lower degree of crystallization, whereas the sharper reflections of NbO-12 indicate more uniform crystallite growth and improved structural ordering. In contrast, the NbO-12 sample exhibits comparatively sharper diffraction peaks and reduced peak broadening relative to NbO-6 and NbO-18, indicating that a 12 h hydrothermal duration promotes a favorable balance between crystallite growth and structural uniformity without inducing excessive grain coarsening, which is further consistent with the uniform morphology observed in FE-SEM images. For NbO-18, although the monoclinic phase is retained, no further significant improvement in diffraction quality is observed, implying that prolonged reaction time does not substantially alter the crystal structure. These observations demonstrate that hydrothermal reaction time plays a crucial role in controlling the crystallization behavior of Nb_2_O_5_ [16,17].

### 4.2. X-Ray Photoelectron Spectroscopy Elucidation

XPS analysis was employed to investigate the surface chemical composition and oxidation states of the Nb_2_O_5_ electrode. The high-resolution Nb 3d spectrum (Figure 2b) exhibits two well-defined peaks located at approximately 207.41 eV and 209.66 eV, which are assigned to the Nb 3d_5/2_ and Nb 3d_3/2_ levels, respectively. The clear spin–orbit splitting of about 2.25 eV is consistent with Nb in the +5-oxidation state, confirming the successful formation of Nb_2_O_5_. The absence of additional shoulders or lower-binding-energy features indicates that reduced niobium species are negligible, reflecting good chemical stability of the oxide [18]. The O 1s spectrum (Figure 2c) further supports this observation and can be resolved into two components. The O 1s spectrum was deconvoluted using a Shirley background and fitted with Gaussian–Lorentzian mixed functions, with consistent FWHM constraints applied to all components to ensure reliable peak fitting. The main peak centered near 530.22 eV corresponds to lattice oxygen bonded within the Nb–O framework, confirming the integrity of the Nb_2_O_5_ crystal structure. The secondary peak appearing at around 531.76 eV is attributed to surface-adsorbed oxygen species or hydroxyl groups, which are commonly introduced during hydrothermal synthesis. The higher-binding-energy O 1s component is attributed to surface hydroxyl or adsorbed oxygen species, which can improve electrolyte wettability and facilitate interfacial ion access during electrochemical operation, rather than implying a universal enhancement of electrochemical activity. The XPS results confirm the presence of Nb^5+^ oxidation states and a stoichiometric Nb–O environment at the surface of the Nb_2_O_5_ samples, consistent with the surface-sensitive nature of the technique. Such surface characteristics are expected to contribute positively to the pseudocapacitive behavior and electrochemical performance of the Nb_2_O_5_ electrode [19].

### 4.3. Morphological and Elemental Compositional Characteristics

**Figure 3:**
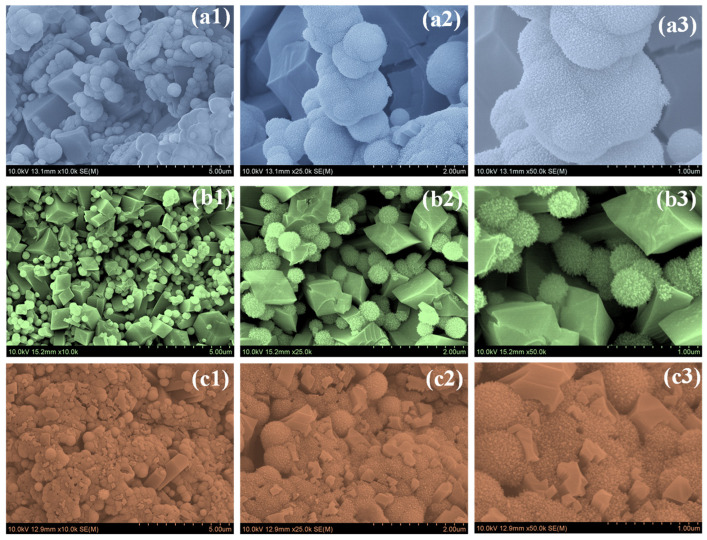
FESEM images of (**a1**–**a3**) NbO-6, (**b1**–**b3**) NbO-12, and (**c1**–**c3**) NbO-18 samples recorded at different magnifications.

Figure 3 shows the FE-SEM images of Nb_2_O_5_ synthesized at different hydrothermal reaction times (NbO-6, NbO-12, and NbO-18) recorded at increasing magnifications. Clear changes in morphology are observed as the reaction time is extended. The NbO-6 sample (Figure 3(a1–a3)) displays a highly nonuniform surface composed of irregular spheres randomly attached to angular, faceted crystallites. The particles are loosely packed and poorly interconnected, with noticeable gaps between neighboring entities. At higher magnification, the surface appears fragmented and lacks a coherent arrangement, indicating that the short reaction time is insufficient to support complete particle growth and structural consolidation. Such a disordered morphology is likely to limit electrolyte accessibility and hinder effective ion transport during electrochemical operation. A markedly different surface structure is observed for the NbO-12 sample (Figure 3(b1–b3)). The particles are more uniformly distributed and organized into spherical aggregates with relatively consistent size. These spheres are constructed from densely packed nanograins, resulting in a rough and textured surface. In addition, the spheres are well integrated with faceted Nb_2_O_5_ crystallites, forming an open and interconnected network. The presence of interparticle voids and open channels suggests improved electrolyte penetration and shortened ion diffusion pathways. This balanced growth behavior indicates that a 12 h reaction time is sufficient to promote controlled nucleation while preventing excessive particle agglomeration. When the reaction time is further increased to 18 h, the NbO-18 sample (Figure 3(c1–c3)) exhibits clear signs of overgrowth. The particles become significantly larger and more compact due to extensive grain fusion. The previously observed ordered features are partially lost, and the structure becomes denser with reduced surface roughness. The close packing of particles and diminished porosity are expected to decrease the number of accessible electroactive sites and restrict ion transport, particularly under high-rate charge–discharge conditions. The FE-SEM observations confirm that hydrothermal reaction time strongly influences the structural evolution of Nb_2_O_5_. Among the three samples, NbO-12 presents the most favorable morphology, combining nanoscale building blocks, well-organized organization, and interconnected porosity. These features are well suited for efficient electrolyte diffusion and rapid charge storage, explaining its superior electrochemical performance in supercapacitor applications [15,20,21]. FE-SEM images recorded at different magnifications reveal that all Nb_2_O_5_ samples consist of nanograin-assembled spherical aggregates coexisting with faceted crystallites. Based on direct estimation from the SEM images, the NbO-6 sample exhibits relatively small and irregular spherical aggregates with diameters typically in the range of ~0.4–0.9 µm. In contrast, the NbO-12 sample shows more uniformly distributed spherical aggregates with sizes predominantly ranging from ~0.6 to 1.2 µm, indicating improved structural uniformity. For the NbO-18 sample, prolonged hydrothermal growth leads to larger and more compact aggregates, with diameters extending beyond ~1.5 µm due to particle coalescence and grain overgrowth. These size ranges are estimated from SEM observations and are intended to provide a semi-quantitative comparison.

**Figure 4:**
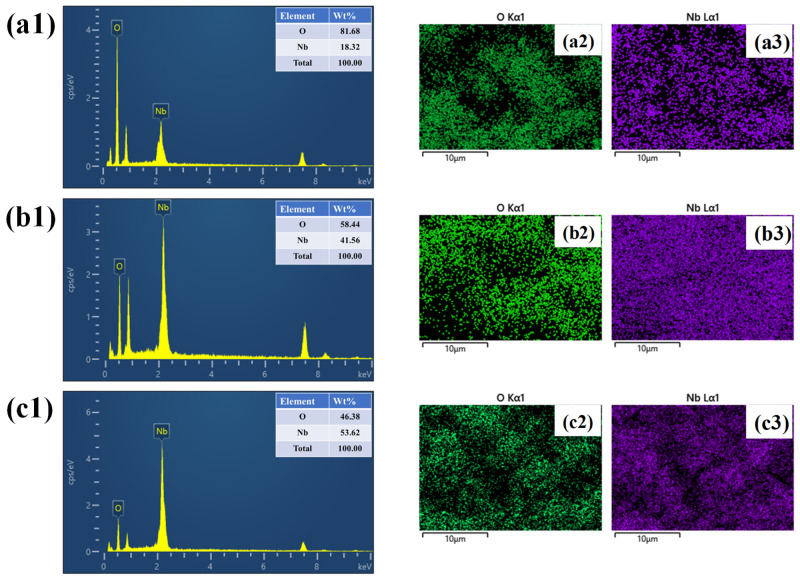
(**a1**–**c1**) EDS spectra, (**a2**–**c3**) elemental mapping images of the NbO-6, NbO-12 and NbO-18 samples.

The EDS spectra shown in (Figure 4(a1–c1)) confirm the presence of only Nb and O in the NbO-6, NbO-12, and NbO-18 samples, respectively, indicating the formation of Nb_2_O_5_ without detectable impurities. The corresponding elemental mapping images for NbO-6, presented in Figure 4(a2,a3), reveal a relatively nonuniform distribution of Nb and O, consistent with the heterogeneous morphology observed in FE-SEM. In contrast, the elemental mappings of NbO-12 shown in Figure 4(b2,b3) display a highly uniform and homogeneous spatial distribution of both elements, reflecting well-controlled growth and an optimized structure. For the NbO-18 sample, the elemental mappings in Figure 4(c2,c3) indicate a denser and more compact distribution of Nb and O, which correlates with particle coarsening and reduced porosity at prolonged reaction time.

### 4.4. Electrochemical Analysis

**Figure 5:**
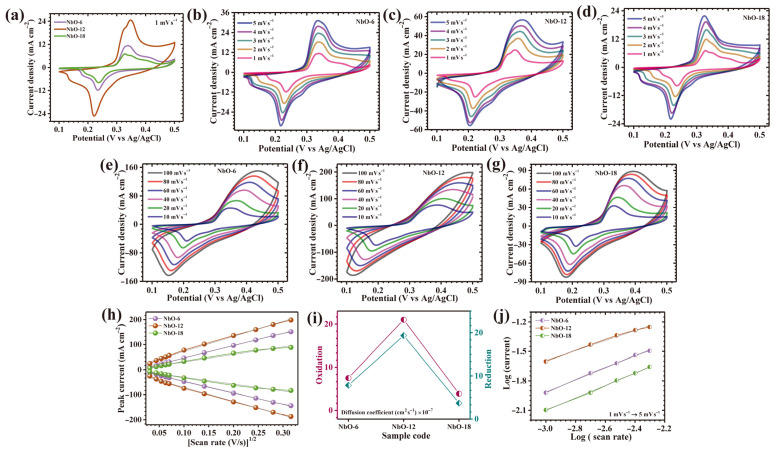
(**a**) Cyclic voltammetry (CV) curves of all Nb_2_O_5_ electrodes at a scan rate of 1 mV s^−1^ in the potential window of 0–0.5 V; CV curves at different scan rates for (**b**–**d**) NbO-6, NbO-12 and NbO-18 at 1–5 mV s^−1^, (**e**–**g**) NbO-6, NbO-12 and NbO-18 at 10–100 mV s^−1^, (**h**) peak current vs. (scan rate)^1/2^ plot; (**i**) diffusion coefficient calculation; (**j**) log(i) vs. log(ν) plot.

The electrochemical behavior of the nanostructured Nb_2_O_5_ electrodes was systematically investigated to elucidate the interplay between time-controlled structural evolution and intrinsic redox activity. Cyclic voltammetry (CV) measurements conducted at a scan rate of 1 mV s^−1^ within a potential range of 0.1–0.5 V (vs. Ag/AgCl) reveal prominent anodic and cathodic redox peaks centered at approximately 0.33 V and 0.21 V, respectively (Figure 5a). These well-resolved and nearly symmetric redox features are indicative of highly reversible pseudocapacitive behavior, originating predominantly from fast faradaic reactions associated with the Nb^5+^/Nb^4+^ redox couple. The electrochemical response suggests an efficient and continuous interaction between niobium active sites and K^+^ ions in the alkaline electrolyte, enabling rapid electron exchange and sustained charge storage kinetics [22]. Among the investigated electrodes, the sample synthesized with a hydrothermal duration of 12 h (NbO-12) displays markedly superior electrochemical characteristics. This enhancement is evidenced by a significantly enlarged CV envelope and intensified redox peak currents, reflecting higher charge storage capacity, accelerated charge propagation, and reduced electrochemical polarization. Such behavior underscores the critical role of precisely regulating synthesis duration in tailoring electroactive architectures. At this optimal growth time, the Nb_2_O_5_ framework develops into a highly cohesive and structurally integrated network that maximizes active surface exposure and promotes facile electrolyte infiltration, thereby strengthening electrode-electrolyte coupling and overall reaction efficiency. Furthermore, the near overlap between anodic and cathodic responses for NbO-12 confirms the quasi-reversible nature of the redox process, suggesting minimal energy loss during cycling and excellent electrochemical stability. The charge storage mechanism governing these electrodes can be attributed to faradaic reactions involving reversible potassium ion insertion and extraction, which may be represented by the following Equation (1) [23]:

(1) $${Nb}_{2}O_{5}+{xK}^{+}+{xe}^{-}⇌{K_{x}Nb}_{2}O_{5}$$

This reaction describes the reversible redox transformation of Nb_2_O_5_ through continuous K^+^ intercalation and de-intercalation within the oxide matrix. The optimized synthesis duration effectively amplifies this cooperative redox activity by enhancing electronic conductivity and reinforcing structural robustness against repeated charge–discharge cycles. To further evaluate electrochemical kinetics and rate performance, CV measurements were performed across a broad range of scan rates. At lower sweep rates (1–5 mV s^−1^; Figure 5b–d), all electrodes exhibit distinct and stable redox peaks, indicating effective faradaic participation throughout the active material. Even at elevated scan rates (10–100 mV s^−1^; Figure 5e–g), the redox features remain clearly discernible, demonstrating rapid reaction kinetics and excellent reversibility. The persistent non-rectangular CV profiles across all scan rates confirm that pseudocapacitive charge storage dominates over pure electric double-layer capacitance. Notably, the intensification of peak currents with increasing scan rate highlights the fast interfacial charge-transfer processes enabled by rational nanostructural engineering. NbO-12 distinctly outperforms the other samples, exhibiting broader CV profiles, stronger redox responses, and enhanced kinetic stability. From a mechanistic perspective, the exceptional electrochemical activity of NbO-12 arises from the precisely tuned synthesis duration of 12 h, which establishes an optimal equilibrium between nucleation and crystal growth. This balance facilitates the formation of homogeneous, porous architectures featuring interconnected channels and accessible voids. In contrast, the NbO-6 electrode, synthesized with a shorter reaction time, suffers from incomplete nucleation and insufficient structural development, resulting in poorly interconnected domains and limited electroactive exposure. Conversely, prolonged hydrothermal treatment in the NbO-18 sample induces excessive grain growth and surface congestion, leading to partial blockage of active sites and restricted ion transport [24].

A detailed assessment of charge-transfer kinetics and ionic diffusion behavior in the Nb_2_O_5_ electrodes was conducted using CV recorded over a broad range of scan rates. As depicted in Figure 5h, both anodic and cathodic peak currents exhibit a strong proportional dependence on the square root of the applied scan rate (*v*^1/2^) for all investigated samples. The presence of this well-defined linear correlation confirms that the electrochemical response of Nb_2_O_5_ electrodes is primarily dictated by diffusion-controlled processes, in which ion migration within the electrode framework governs the overall reaction rate rather than surface-limited capacitive effects. This behavior suggests that the redox reactions are closely coupled with the transport of electrolyte ions across the electrode-electrolyte interface and into the bulk of the active material. To quantitatively evaluate the diffusion characteristics of the electroactive species, the apparent diffusion coefficients (*D*) were calculated using the classical Randles-Sevcik relation, expressed as Equation (2) [25]:

(2) $$D^{1/2}=\frac{i_{p}}{2.69\times 10^{5}\times n^{3/2}\times A\times C\times v^{1/2}}$$

where *i_p_* denotes the peak current obtained from CV curves, *n* represents the number of electrons participating in the redox reaction, *A* corresponds to the electrochemically active surface area of the electrode, *C* is the concentration of electroactive species in the electrolyte, and *v* refers to the scan rate. Using this formalism, diffusion coefficients were determined at a fixed scan rate of 10 mV s^−1^ to ensure consistent and reliable comparison among the samples. The calculated diffusion coefficients are summarized in Table 1, and their comparative distribution is illustrated in Figure 5i. Among all electrodes, the NbO-12 sample exhibits the highest diffusion coefficient, indicating substantially enhanced ion transport and more efficient charge-transfer dynamics. In contrast, electrodes synthesized under non-ideal reaction durations show diminished diffusion characteristics. The NbO-18 electrode presents the lowest diffusion coefficient, primarily due to excessive structural growth and agglomeration that reduce accessible electroactive sites and hinder ion diffusion through blocked or narrowed transport channels.

The intrinsic charge storage mechanism of the Nb_2_O_5_ electrodes was further elucidated through a quantitative kinetic analysis based on the power-law relationship between peak current response (*i_p_*) and the applied scan rate (*v*), expressed as Equation (3) [26]:

(3) $$i={av}^{b}$$

In this relation, the exponent *b* serves as a decisive kinetic indicator that reflects the dominant electrochemical process. Specifically, a *b* value approaching 0.5 corresponds to diffusion-limited faradaic behavior, whereas values trending toward 1 signify surface-controlled capacitive storage. To determine the *b*-values, logarithmic plots of peak current versus scan rate were plotted, as shown in Figure 5j, and linear fitting was employed to extract accurate slopes. For all Nb_2_O_5_ electrodes, the derived *b*-values fall within a narrow range of 0.51–0.63 (Table 1), clearly confirming that ion-diffusion-controlled redox reactions constitute the primary charge storage pathway, accompanied by a secondary but non-negligible capacitive contribution.

To achieve a more refined distinction between surface-dominated capacitive processes and diffusion-governed faradaic reactions, the current response at each potential was further deconvoluted using the following kinetic Expression (4) [11]:

(4) $$i\left(V\right)=k_{1}v{+k_{2}v}^{1/2}$$

**Figure 6:**
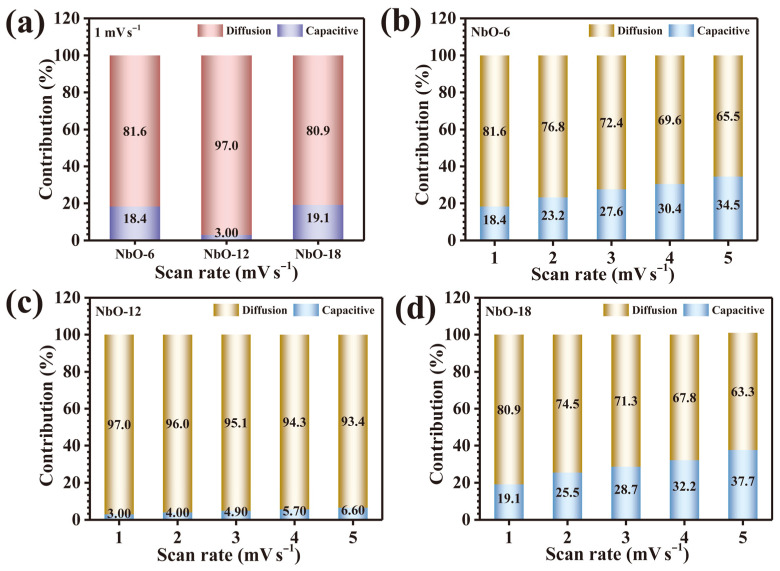
Capacitive and diffusion-controlled charge-storage contributions: (**a**) comparison of all Nb_2_O_5_ electrodes at 1 mV s^−1^, (**b**) NbO-6, (**c**) NbO-12, and (**d**) NbO-18 at various scan rates.

Here, the term *k*_1_*v* represents capacitive charge storage arising from rapid surface reactions at the electrode-electrolyte interface, while *k*_2_*v*^1/2^ accounts for diffusion-controlled contributions associated with ion transport into redox-active regions of the electrode. By plotting *i*(*V*)/*v*^1/2^ vs. *v*^1/2^, the constants *k*_1_ and *k*_2_ were accurately determined, enabling quantitative separation of the two charge storage components across the entire potential window. Consequently, the total stored charge can be expressed as the sum of surface-controlled and diffusion-controlled contributions according to Equation (5) [11]:

(5) $$Q_{t}=Q_{s}+Q_{d}$$

where *Q_s_* corresponds to capacitive storage and *Q_d_* represents diffusion-dominated faradaic storage. At a representative low scan rate of 1 mV s^−1^, the extracted charge contributions reveal a pronounced dominance of diffusion-controlled processes for all Nb_2_O_5_ electrodes. Specifically, the capacitive-to-diffusion ratios were determined to be 18.4/81.6%, 3.0/97.0%, and 19.1/80.9% for NbO-6, NbO-12, and NbO-18, respectively, as illustrated in Figure 6a. Notably, the NbO-12 electrode exhibits the most pronounced diffusion-dominated behavior, with nearly 97% of the stored charge arising from faradaic ion diffusion, signifying exceptional ionic mobility and accelerated electrochemical kinetics. This outstanding performance is mechanistically attributed to its organized porous architecture featuring interconnected void channels, which ensure efficient electrolyte infiltration and facilitate deep ion penetration into the electroactive Nb_2_O_5_ matrix. Furthermore, the evolution of charge storage behavior with increasing scan rate (1–5 mV s^−1^) was systematically analyzed for all electrodes. As depicted in Figure 6b–d, a gradual increase in capacitive contribution is observed across the NbO-6, NbO-12, and NbO-18 samples as the scan rate increases. This trend arises from kinetic constraints on ion diffusion under rapid potential sweeps, wherein limited diffusion lengths favor surface-localized capacitive processes over bulk diffusion-controlled reactions [21]. Despite this inevitable shift, the NbO-12 electrode consistently retains the highest fraction of diffusion-governed storage even at elevated scan rates, highlighting its superior structural adaptability in mitigating mass-transfer limitations. Collectively, this comprehensive kinetic analysis clearly demonstrates that precise control of hydrothermal reaction time plays a pivotal role in dictating the mechanistic balance between capacitive and diffusion-controlled charge storage. The optimized NbO-12 electrode achieves an exceptional synergy between structural design and redox kinetics, resulting in dominant faradaic behavior.

**Figure 7:**
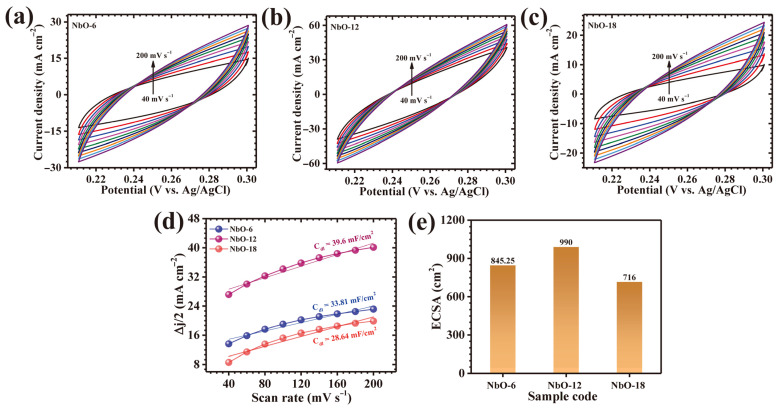
(**a**–**c**) CV curves of the three electrodes recorded at different scan rates within the non-faradaic potential window, (**d**) linear fitting of current response versus scan rate for extracting the double-layer capacitance (Cdl), and (**e**) calculated electrochemically active surface area (ECSA) values of the electrodes.

The electrochemically accessible surface area of the NbO-6, NbO-12, and NbO-18 electrodes was estimated by analyzing their capacitive behavior in a non-faradaic region. For this purpose, scan-rate-dependent CV curves were collected within a potential window where no redox activity occurs (Figure 7a–c), ensuring that the measured current originates mainly from interfacial double-layer charging rather than faradaic reactions. The charging current response increased systematically with scan rate, and linear fitting of this dependence enabled extraction of the double-layer capacitance (Cdl) (Figure 7d). The ECSA was then determined using Equation (6) [15]:

(6) $$ECSA=\frac{c_{dl}}{C_{s}}$$

where Cs corresponds to the specific capacitance of a smooth reference surface in 1 M KOH (0.04 mF cm^−2^). Using this method, the calculated ECSA values were ~845.25 cm^2^ (NbO-6), ~990 cm^2^ (NbO-12), and ~716 cm^2^ (NbO-18) (Figure 7e). The significantly higher ECSA of NbO-12 indicates the presence of a greater number of electrolyte-accessible active sites, which is consistent with its porous interconnected framework and explains its enhanced charge-storage performance.

**Figure 8:**
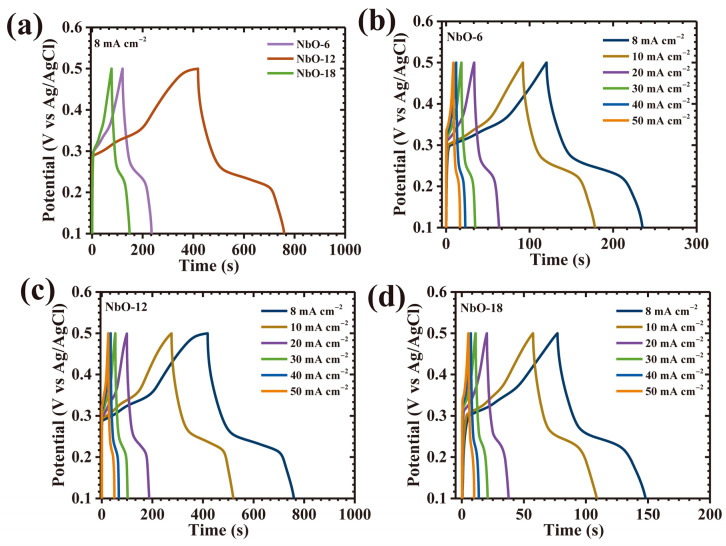
(**a**) GCD curves of all Nb_2_O_5_ electrodes at 8 mA cm^−2^; GCD profiles at various current densities for (**b**) NbO-6, (**c**) NbO-12, and (**d**) NbO-18 at various scan rates.

**Figure 9:**
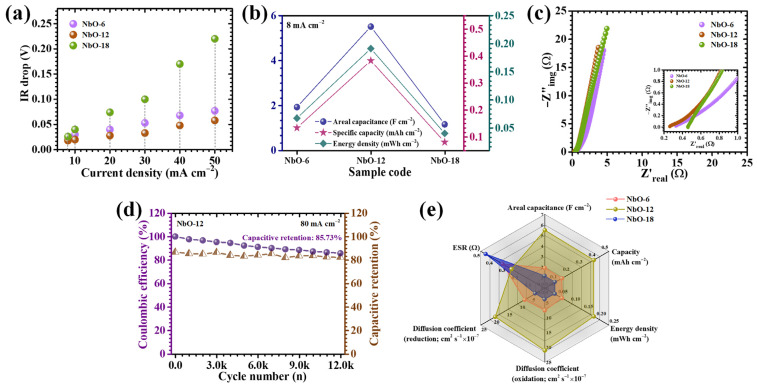
(**a**) IR-drop analysis and comparison of areal capacitance, specific capacity, and energy density for Nb_2_O_5_ electrodes; (**b**) Comprehensive electrochemical performance metrics of Nb_2_O_5_ electrodes (**c**) Nyquist (EIS) plots of the electrodes; (**d**) long-term cycling stability of the Nb_2_O_5_ electrode over 12,000 GCD cycles (**e**) Radar plot.

The galvanostatic charge–discharge (GCD) response of the Nb_2_O_5_ electrodes was systematically examined to assess their charge storage efficiency, rate capability, and current-dependent electrochemical behavior. Figure 8a presents a direct comparison of GCD profiles recorded at a fixed current density of 8 mA cm^−2^ within a potential window of 0.1–0.5 V, clearly revealing pronounced distinctions among the three electrodes. To further elucidate rate-dependent characteristics, GCD measurements were performed over a wide current density range from 8 to 50 mA cm^−2^ for NbO-6, NbO-12, and NbO-18 electrodes, as illustrated in Figure 8b–d. Across all samples, the charge–discharge curves deviate markedly from ideal triangular shapes and exhibit evident voltage plateaus during discharge, confirming that the charge storage mechanism is dominated by diffusion-controlled faradaic redox reactions. Such nonlinear profiles are characteristic of battery-type pseudocapacitive behavior arising from reversible ion intercalation and redox transitions within the Nb_2_O_5_ lattice [27]. Among the three electrodes, NbO-12 displays the most pronounced nonlinearity accompanied by a smooth and gradual potential decay, indicative of highly efficient pseudocapacitive activity supported by rapid ion transport and reversible redox processes [28]. In addition, the NbO-12 electrode exhibits a substantially prolonged discharge duration relative to NbO-6 and NbO-18 at identical current densities, directly reflecting its superior charge storage capacity and enhanced utilization of electroactive sites. The nearly symmetric charge-discharge profiles observed for all electrodes further indicate high coulombic efficiency and excellent electrochemical reversibility, implying stable ion diffusion dynamics and minimal polarization during repeated cycling. Interestingly, the NbO-12 electrode demonstrates the smallest instantaneous voltage drop (IR-drop) at the onset of discharge, together with an almost perfectly symmetric GCD profile. The variation in IR-drop as a function of applied current density, shown in Figure 9a, reveals a consistent decrease in IR-drop with lowering current density for all samples, confirming reduced ohmic losses and improved interfacial contact between electrode and electrolyte. Despite the low IR-drop observed for NbO-18, the NbO-12 electrode consistently exhibits the smallest overall IR-drop across the entire current density range, underscoring its optimized charge transport pathways and superior electrochemical kinetics.

To quantitatively evaluate the electrochemical performance, the areal capacitance (C_A_), energy density (ED), and power density (PD) were calculated using formulations specifically adapted for nonlinear GCD profiles, as given by Equations (7)–(9) [29,30]:

(7) $$C_{A}=\frac{I\times 2\times \int V\left(t\right)dt}{A\times {\left(∆V\right)}^{2}}$$

(8) $$ED=\frac{1}{2\times 3600}\;C_{A}\times {dV}^{2}$$

(9) $$PD=\frac{ED\times 3600}{T_{d}}$$

where *I* represents the discharge current, ∫*V*(*t*)*dt* denotes the time-integrated discharge potential accounting for non-ideal pseudocapacitive behavior, *A* is the electrochemically active surface area, Δ*V* corresponds to the applied potential window, and *T_d_* is the discharge time. This calculation methodology is particularly essential for pseudocapacitive electrodes, in which faradaic reactions produce nonlinear discharge characteristics that cannot be accurately described using conventional linear capacitance models. At a current density of 8 mA cm^−2^, the calculated areal capacitances were 1.92 F cm^−2^ for NbO-6, 5.504 F cm^−2^ for NbO-12, and 1.152 F cm^−2^ for NbO-18, as summarized in Table 2 and depicted in Figure 9b. Among all compositions, NbO-12 delivers the highest capacitance, directly validating the structural and interfacial advantages imparted by the optimized hydrothermal reaction duration. This enhanced charge storage performance originates from multiple synergistic factors, including an enlarged electrochemically active surface exposing abundant redox-active Nb sites, improved electronic conductivity through a well-connected Nb_2_O_5_ network that minimizes charge-transfer resistance, and accelerated ion diffusion enabled by open, interconnected pore channels facilitating deep electrolyte penetration. The synthesis time plays a decisive role in preserving this favorable architecture by regulating nucleation and growth kinetics [31]. Specifically, it prevents excessive particle agglomeration and inhibits the restacking of spheres and rectangular rods, thereby maintaining a porous framework essential for rapid ionic motion and efficient faradaic activity. As expected, all electrodes exhibit a gradual decline in areal capacitance and energy density with increasing current density (Table 2), primarily due to limited ion diffusion and reduced accessibility of deeper electroactive sites under high-rate operation. Nevertheless, the NbO-12 electrode demonstrates outstanding rate capability, retaining approximately 44% of its initial capacitance even at a high current density of 50 mA cm^−2^. This remarkable retention highlights its robust structural stability, efficient ion transport pathways, and strong resistance to kinetic degradation under demanding operating conditions.

Electrochemical impedance spectroscopy (EIS) was employed to gain deeper insight into the charge-transfer characteristics, internal resistance, and ion diffusion behavior of the Nb_2_O_5_ electrodes synthesized at different hydrothermal durations. The measurements were conducted over a wide frequency range (0.1 k to 10 k), and the resulting Nyquist plots are presented in Figure 9c. These plots, representing the relationship between the real (Z′) and imaginary (−Z″) components of impedance, provide comprehensive information regarding both resistive and capacitive elements governing the electrochemical kinetics of the electrodes. In the high-frequency region, the intercept of the Nyquist plot with the real axis corresponds to the equivalent series resistance (ESR), which arises from the combined contributions of electrolyte resistance, intrinsic resistance of the active material, and contact resistance at the electrode-current collector interface [32]. As summarized in Table 1, the NbO-12 electrode exhibits the lowest ESR of 0.26 Ω among all samples, indicating superior electrical conductivity and more efficient ionic transport pathways. The semicircular region observed at intermediate frequencies is related to the charge-transfer resistance, reflecting the kinetics of faradaic redox reactions at the electrode-electrolyte interface. Notably, the NbO-12 electrode displays the smallest semicircle diameter, signifying accelerated electron-transfer kinetics and reduced interfacial barriers. This reduced resistance is directly associated with the optimized nanostructure formed at the 12 h hydrothermal duration, which ensures intimate contact between Nb_2_O_5_ domains and the conductive substrate while maintaining open channels for electrolyte access.

To assess long-term electrochemical durability, galvanostatic cycling stability tests were conducted on the NbO-12 electrode at a high current density of 80 mA cm^−2^ (Figure 9d) for 12,000 cycles. The electrode demonstrates excellent cycling endurance, retaining 85.73% of a substantial fraction of its initial capacitance, while maintaining a high coulombic efficiency of 82.17%, after prolonged charge–discharge operation. This remarkable stability reflects the robust structural integrity and reversible redox behavior of the Nb_2_O_5_ framework. The superior cycling performance is primarily attributed to the hierarchically organized porous architecture developed at the optimized synthesis duration, which effectively accommodates volume changes associated with repeated K^+^ insertion and extraction without inducing structural collapse or loss of electrical connectivity. The interconnected pore network and mechanically stable framework suppress stress accumulation during cycling, preserve electrode-electrolyte interfacial integrity, and ensure sustained ion diffusion throughout extended operation [33]. The slight capacitance decay (14.27%) observed after long-term cycling can be ascribed to gradual ion trapping within deep or less accessible microporous regions, as well as minor structural rearrangements at the electrode surface, which marginally limit the reversibility of faradaic reactions. Nevertheless, the high retention and stable electrochemical response confirm the excellent durability of the NbO-12 electrode under demanding operational conditions.

A radar plot was constructed (Figure 9e), to provide a complete comparison of electrochemical performance incorporating key parameters such as areal capacitance, energy density, diffusion coefficient, and equivalent series resistance for NbO-6, NbO-12, and NbO-18 electrodes. This multidimensional visualization clearly highlights the superior and well-balanced performance of the NbO-12 electrode across all evaluated metrics. The expanded and uniform profile of NbO-12 reflects its optimal integration of high charge storage capability, rapid ion transport, and minimal internal resistance, collectively confirming the effectiveness of hydrothermal time optimization in engineering high-performance Nb_2_O_5_ electrodes. In contrast, electrodes synthesized at non-ideal durations exhibit compromised performance due to structural imbalance. Overall, the radar plot analysis reinforces that the 12 h hydrothermal duration represents an optimal synthesis window, enabling the formation of a structurally robust, electrochemically efficient Nb_2_O_5_ architecture well-suited for advanced supercapacitor applications.

## 5. Electrochemical Performance of Asymmetric Supercapacitor Device

**Figure 10:**
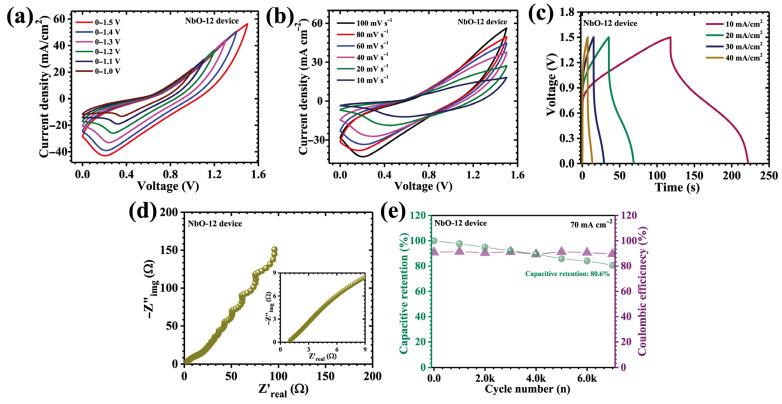
(**a**) CV curves of the NbO-12//AC hybrid device recorded within potential windows ranging from 0–1.0 V to 0–1.5 V; (**b**) CV curves of the device at scan rates from 10 to 100 mV s^−1^ (0–1.5 V); (**c**) GCD curves at different current densities; (**d**) EIS analysis; (**e**) long-term cycling stability over 7000 GCD cycles.

To bridge the gap between fundamental electrode evaluation and practical energy storage application, an asymmetric pouch-type supercapacitor device (APSD) was assembled using the optimized NbO-12 electrode as the positive terminal and activated carbon (AC) as the negative terminal. The selection of AC as the counter electrode is strategically motivated by its excellent electric double-layer capacitance, which complements the diffusion-controlled pseudocapacitive behavior of Nb_2_O_5_. This asymmetric configuration enables effective voltage expansion while maintaining balanced charge storage kinetics, thereby enhancing overall device performance. Both electrodes were fabricated on nickel foam current collectors to ensure high electrical conductivity, mechanical robustness, and reliable interfacial contact. For the preparation of the negative electrode, a uniform slurry was formulated by mixing activated carbon with acetylene black as a conductive additive and polyvinylidene fluoride (PVDF) as a binder, using N-methyl-2-pyrrolidone (NMP) as the solvent. The mixture was thoroughly homogenized to achieve uniform dispersion and then evenly coated onto the nickel foam substrate (2 mg cm^−2^). The coated electrodes were subsequently dried at 60 °C for 12 h to ensure complete solvent evaporation and strong adhesion of the active material layer. The asymmetric pouch cell was assembled using a filter paper separator soaked in 2 M KOH aqueous electrolyte, which simultaneously served as the ionic medium and physical barrier between the electrodes. The device was sealed to prevent electrolyte leakage, evaporation, and atmospheric contamination, thereby ensuring electrochemical stability during extended operation. Electrochemical characterization of the APSD was systematically carried out using CV, GCD, and EIS. The asymmetric device was designed based on the charge balance principle (*q^+^* = *q^−^*), where q=C×∆V×m. In this work, equal areal mass loadings were employed for NbO-12 and AC electrodes, and the suitability of this ratio was validated through stable CV/GCD characteristics and cycling performance without signs of electrode over-polarization. All device-level areal electrochemical metrics were calculated based on the geometric device overlap area (i.e., the effective total device area). The NbO-12 electrode demonstrated stable electrochemical operation within a potential window of 0.1–0.5 V (vs. Ag/AgCl), whereas the activated carbon (AC) electrode exhibited reliable capacitive behavior in the range of −1.0 to 0 V. The complementary voltage stability of the two electrodes enabled the NbO-12//AC asymmetric device to operate safely over an extended cell voltage of up to 1.5 V without inducing electrolyte decomposition or electrode instability. To validate the electrochemical robustness of the assembled device, CV measurements were conducted over progressively different voltage windows from 1.0 to 1.5 V, as shown in Figure 10a. The resulting CV curves consistently display well-defined pseudocapacitive features accompanied by stable current responses, confirming the excellent reversibility and structural integrity of the device across the entire operating voltage range. Further, the CV curves of the NbO-12//AC device, recorded over scan rates ranging from 10 to 100 mV s^−1^ (Figure 10b), exhibit quasi-rectangular profiles accompanied by discernible redox humps. This behavior confirms the coexistence of surface-controlled capacitive charge storage contributed by the AC electrode and diffusion-dominated faradaic reactions originating from the Nb_2_O_5_ electrode. Notably, the device operates stably over an extended potential window of 1.5 V, which is relatively wide for an aqueous electrolyte system. This voltage expansion is enabled by the synergistic coupling of the high redox activity of NbO-12 with the large surface area and electrochemical stability of the AC electrode, allowing efficient utilization of both faradaic and non-faradaic storage mechanisms.

The corresponding GCD profiles (Figure 10c) further validate the hybrid charge storage behavior of the APSD, displaying nonlinear discharge curves characteristic of pseudocapacitive systems. At a current density of 10 mA cm^−2^, the device delivers a high areal capacitance of 0.324 F cm^−2^, along with an energy density of 0.101 mWh cm^−2^ and a power density of 1.74 mW cm^−2^ (Table 3). These performance metrics demonstrate an effective balance between energy and power delivery, arising from the complementary interaction between the Nb_2_O_5_ positive electrode and the AC negative electrode within the asymmetric configuration. EIS measurements (Figure 10d) provide further insight into the charge transport characteristics and interfacial dynamics of the assembled device. The extracted ESR of approximately 1.04 Ω confirms the excellent electrical conductivity and rapid ionic transport within the device. This low resistance is directly attributed to the improved nanostructure of the NbO-12 electrode, which provides continuous electron transport pathways and open ion diffusion channels, as well as to the effective electrode-electrolyte interface established in the pouch configuration. The long-term durability of the APSD was evaluated through galvanostatic cycling stability tests performed at a current density of 70 mA cm^−2^ for 7000 continuous charge–discharge cycles (Figure 10e). Remarkably, the device retains 80.6% of its initial capacitance while maintaining a high coulombic efficiency of 89.47%, indicating excellent reversibility and electrochemical stability. The coulombic efficiency value is mainly associated with polarization losses and minor parasitic interfacial processes typical of aqueous hybrid pouch-type devices operated at wide voltage windows, while the stable cycling retention confirms reliable electrochemical reversibility. This robust cycling performance is primarily attributed to the structurally stable and porous architecture of the NbO-12 electrode, which effectively accommodates volume variations associated with repeated ion insertion and extraction. The interconnected framework preserves electrical contact and interfacial integrity during prolonged cycling, thereby mitigating performance degradation. Overall, the NbO-12//AC asymmetric pouch-type supercapacitor demonstrates a compelling combination of high areal capacitance, wide operating voltage window, low internal resistance, and outstanding cycling durability. These results highlight the effectiveness of hydrothermal reaction-time optimization in engineering Nb_2_O_5_ electrodes with superior device-level performance.

## 6. Conclusions

This study demonstrates that hydrothermal reaction-time engineering provides an effective route to tailor Nb_2_O_5_ nanostructures and directly regulate their electrochemical charge-storage behavior. Notably, hydrothermal reaction-time modulation represents a simple, reproducible, and scalable synthesis parameter, offering practical feasibility for large-area Nb_2_O_5_ electrode fabrication without altering composition or requiring complex processing. Among the synthesized electrodes, NbO-12 achieved an optimized nanograin-assembled porous framework that maximizes electroactive accessibility while maintaining efficient ion-diffusion pathways, resulting in markedly improved areal capacitance and rate capability of 5.504 F cm^−2^ at 8 mA cm^−2^, outperforming NbO-6 (1.92 F cm^−2^) and NbO-18 (1.152 F cm^−2^). Mechanistic evaluation confirms predominantly diffusion-controlled pseudocapacitive storage with reduced resistance characteristics, highlighting the kinetic advantages enabled by the optimized microstructure. This indicates efficient utilization of diffusion-accessible redox sites at low scan rates, while the optimized porous framework minimizes diffusion limitations and thereby supports strong rate performance at higher scan rates. Importantly, NbO-12 exhibited excellent durability with strong capacitance retention over extended cycling, reflecting robust structural stability under repetitive electrochemical operation. Beyond electrode-level performance, the NbO-12//AC asymmetric pouch-type supercapacitor delivered stable operation within a widened 1.5 V window with competitive areal energy storage output and reliable cycling durability. As a general limitation of diffusion-involved pseudocapacitive systems, performance may decline at very high current densities due to polarization and ion-transport constraints; thus, further optimization of electrode architecture could enhance ultra-high-rate operation. Overall, these findings validate hydrothermal time modulation as a scalable and practically relevant strategy for designing high-performance Nb_2_O_5_-based electrodes and aqueous asymmetric supercapacitors for next-generation energy storage applications.

## Figures and Tables

**Table 1:** Estimated Diffusion coefficient, b-values, and series resistance values of NbO-6, NbO-12 and NbO-18 samples.

Sample Code	Diffusion Coefficient (cm^2^/s) × 10^−7^		b-Value	ESR (Ω)
	Oxidation	Reduction		
NbO-6	7.5	7.83	0.61	0.33
NbO-12	21.02	19.33	0.51	0.26
NbO-18	3.87	3.71	0.63	0.46

**Table 2:** Evaluation of calculated areal capacitance, specific capacity, energy density, and power density values of NbO-6, NbO-12 and NbO-18 electrodes.

Sample Code	I (mA cm^−2^)	Areal Capacitance C_A_ (F cm^−2^)	Capacity (mAh cm^−2^)	Energy Density ED (mWh cm^−2^)	Power Density PD (mW cm^−2^)
NbO-6	8	1.920	0.133	0.067	2.03
	10	1.760	0.122	0.061	2.47
	20	1.216	0.084	0.042	4.61
	30	0.984	0.068	0.034	6.83
	40	0.864	0.060	0.030	9.23
	50	0.800	0.056	0.028	12.50
NbO-12	8	5.504	0.382	0.191	1.95
	10	5.040	0.350	0.175	2.46
	20	3.520	0.244	0.122	4.54
	30	2.880	0.200	0.100	6.92
	40	2.560	0.178	0.089	9.41
	50	2.400	0.167	0.083	12.00
NbO-18	8	1.152	0.080	0.040	1.95
	10	1.040	0.072	0.036	2.36
	20	0.720	0.050	0.025	4.50
	30	0.600	0.042	0.021	6.82
	40	0.480	0.033	0.017	8.57
	50	0.440	0.031	0.015	11.00

**Table 3:** Calculated energy storage parameters of NbO-12//AC asymmetric pouch-type supercapacitor device.

Sample Code	I (mA)	CA (F cm^−2^)	C (mAh cm^−2^)	ED (mWh cm^−2^)	PD (mW cm^−2^)
NbO-12 device	10	0.324	0.068	0.101	1.74
	20	0.154	0.032	0.048	2.54
	30	0.076	0.016	0.024	3.05
	40	0.036	0.007	0.011	3.33

## Data Availability

The data presented in this study are available on request from the corresponding author.
